# Visualization-Based Rapid Screening and Quantitative Analysis of Target Peptides for Meat Authentication

**DOI:** 10.3390/foods14173048

**Published:** 2025-08-29

**Authors:** Yingying Zhang, Chaodi Kang, Mengyao Liu, Siyu Jiang, Yingying Li, Wenping Guo, Weiheng Kong, Shouwei Wang

**Affiliations:** 1 China Meat Research Center, Beijing 100068, China; yingying_sys@163.com (Y.Z.);; 2 Science and Technology Research Center of China Customs, Beijing 100026, China

**Keywords:** quantitative peptide screening, meat authentication, multivariate statistical analysis, mass, species-specific markers

## Abstract

Amidst growing demand for meat products, concerns regarding their authenticity and safety have intensified, primarily due to potential fraudulent substitutions of cheaper meats, which are not accurately labeled. This study presents a novel strategy for the rapid screening and validation of target peptides for accurate quantitative analysis using high-resolution mass spectrometry (HRMS) coupled with multivariate statistical analysis. By integrating hierarchical clustering analysis (HCA) with parallel reaction monitoring (PRM), five species-specific peptides were validated as reliable biomarkers for pork quantification. These peptides demonstrated accurate quantification in simulated meat products with known accurate contents, achieving recoveries of 78–128%, with RSD less than 12%. This methodology markedly enhances screening efficiency by excluding 80% of non-quantitative peptides, providing a robust solution for meat authenticity verification.

## 1. Introduction

The authenticity and safety of meat products constitute a persistent global challenge, particularly given the escalating risks of adulteration and mislabeling in increasingly complex food supply chains [1,2,3]. Quantitative determination of meat content in processed products has substantial market relevance, particularly as modern manufacturing increasingly utilizes hybrid meat formulations rather than single-species materials. Despite regulatory labeling requirements specifying ingredients, the precise proportion of each meat species remains unverified, constituting a critical challenge for current food surveillance systems.

Current methodologies for detecting adulteration and quantifying meat content in processed products encompass spectroscopic techniques, DNA-based assays, and protein-based analytical approaches.

Spectroscopic techniques have gained extensive adoption in food authentication due to their non-destructive nature, rapid analysis capability and real-time monitoring potential, such as near-infrared (NIR) spectroscopy [4], mid-infrared (MIR) spectroscopy, Raman spectroscopy [5], nuclear magnetic resonance (NMR) [6], fluorescence spectroscopy [7,8], laser-induced breakdown spectroscopy (LIBS) [9], and hyperspectral imaging (HSI) [10]. Nevertheless, critical limitations persist, including interference from complex sample matrices, spectral overlapping and insufficient specificity, limited sensitivity for low-concentration adulterants and poor model generalizability across diverse sample sets [11].

DNA-based detection techniques have served as the gold standard for meat authentication due to their high specificity and analytical stability, such as real-time polymerase chain reaction (RT-PCR) [12], digital polymerase chain reaction (dPCR), DNA barcoding method [13], loop-mediated isothermal amplification (LAMP) [14], and the biosensor method. Nevertheless, inherent limitations exist, including matrix interference and variable DNA extraction efficiency, amplification biases, sample heterogeneity issues coupled with sampling bias and degradation of DNA integrity during thermal processing [15].

Protein-based analytical techniques encompass enzyme-linked immunosorbent assays (ELISA) [16], sodium dodecyl sulfate–polyacrylamide gel electrophoresis (SDS-PAGE) [17], and mass spectrometry (MS) [18]. While ELISA suffers from false-positive signals, poor reproducibility, poor sensitivity to processing treatments, and limited specificity, SDS-PAGE exhibits low resolution, insufficient specificity, inadequate sensitivity, limited qualitative/quantitative capability, and poor compatibility with complex matrices [19]. Conversely, MS has emerged as a premier analytical technology owing to its superior reproducibility, high sensitivity/specificity, capacity for simultaneous quantification of species-specific markers and discrimination of tissue origins within a single species for meat adulteration detection [20].

While species identification has been extensively studied, quantitative analysis of meat adulteration [21,22] remains underdeveloped, which also plays a crucial role in the analysis of meat products. Prandi [23] identified species-specific marker peptides for eight kinds of meats (duck, rabbit, chicken, turkey, buffalo, horse, deer, and sheep) based on mass spectrometry. By establishing calibration curves, the meat content was determined and applied to complex food matrices (Bolognese sauce). The method has good sensitivity (detection limit of 0.2–0.8%). Sentandreu [24] found that the DQGTFEDFVGLR and ALGQNPTNAEINK peptides from chicken myosin light chain 3 were specific peptide markers. Through the use of isotope-labeled (Absolute Quantification with Stable Isotopes, AQUA) peptides (biomarker peptides DQGTFEDF-(^13^C_9_,^15^N)VEGLR and AL(^13^C_6_,^15^N)GQNPTNAEINK), the quantitative analysis of chicken was achieved.

The integration of proteomics with multivariate statistical analysis has further enhanced the capabilities of MS in food authentication [25,26]. Windarsih [25] used untargeted proteomics and metabolomics by liquid chromatography–high resolution mass spectrometry (LC-HRMS) and chemometrics of principal component analysis (PCA) and partial least square-discriminant analysis (PLS-DA) to differentiate authentic and adulterated pangasius hypopthalmus meat (PHM) successfully. Our team previously utilized multivariate statistical analysis, including PCA and OPLS-DA, to distinguish meat products containing different concentrations of fox meat, demonstrating that these peptides of different content samples could be effectively separated without interference, that confirmed the feasibility of quantitative analysis for fox meat content by using species-specific peptides [27].

Species-specific quantitative peptides are the key to accurate quantitative analysis. However, current peptide targeting strategies persist in database-dependent identification of species-specific peptides from sample-derived MS data, followed by recovery rate validation of candidate peptides to confirm quantitative suitability. However, due to the enormous number of peptide-spectrum matches, performing uniqueness queries peptide-by-peptide against the entire database is labor-intensive and inefficient. Herein, a novel hierarchical clustering-driven workflow that implements positive correlation-based pre-screening prior to species-specificity verification was proposed. This strategy achieves 80% elimination of non-informative peptide signals and accelerates processing efficiency.

## 2. Materials and Methods

### 2.1. Materials and Reagents

The materials and reagents used in this study are similar to those employed in our previous research [28]. Trypsin (BioReagent), dithiothreitol (DTT), and iodoacetamide (IAA) were from Sigma-Aldrich Corporation (St. Louis, MO, USA). Formic acid (FA), acetic acid, and acetonitrile (ACN) (LC grade) were from Anpel Laboratory Technologies Inc. (Shanghai, China). Urea, thiourea, hydrochloric acid (HCl), and Tris (hydroxymethyl) aminomethane (Tris) (analytical grade) were from Sinopharm Chemical Reagent Co., Ltd. (Shanghai, China). C_18_ solid-phase extraction column (60 mg, 3 mL) was from Waters Corporation (Milford, MA, USA). Ultra-pure Water was Wahaha purified water, purchased from local markets.

### 2.2. Sample Preparation

Fresh pork and beef were purchased from the local markets.

Simulated meatball samples containing specific amounts of pork were prepared according to the meatball process. Pork, beef, and various food additives (including sodium tripolyphosphate, sodium pyrophosphate, sodium hexametaphosphate, sodium erythorbate, carrageenan, salt, sugar, garlic powder, pepper powder, ginger powder, red wine, monosodium glutamate, and vegetable oil) were mixed in specific proportions to prepare four groups of samples with different pork contents (12.75 g/100 g, 36.95 g/100 g, 52.24 g/100 g, and 76.45 g/100 g). Each group of samples was shaped into about 10 g meatballs, which were then boiled for 15 min in a water bath. Then, the meatballs were removed and cooled to room temperature. Subsequently, all meatballs from the same group were homogenized to ensure uniform mixing. The homogenized samples were stored at −18 °C.

### 2.3. Sample Pretreatment

Sample pretreatment followed the protocol described by our previous article [28], with minor modifications as detailed below. All samples underwent six biological parallel tests.

Extraction: A 2 g sample was weighed in a plastic centrifuge tube. A total of 20 mL extraction solution (Tris-HCl 0.05 M, urea 7 M, thiourea 2 M, pH 8.0), pre-cooled in advance, was added and homogenized in an ice-water bath. Then, the extract was centrifuged at 12,000 rpm for 20 min at 4 °C.

Digestion: A total of 200 μL of the supernatant was pipetted in a 5 mL plastic centrifuge tube and reacted in a water bath at 56 °C for 60 min with 30 μL of 0.1 M DTT solution. After cooling to room temperature, the alkylation reaction was carried out with 30 μL of 0.1 M IAA solution in the dark at room temperature for 30 min. Subsequently, 1.8 mL of Tris-HCl buffer (25 mM Tris-HCl, pH 8.0) was added, followed by the addition of 60 µL of 1.0 mg/mL trypsin solution, and the mixture was incubated at 37 °C for overnight. Then, the reaction was terminated by adding 15 µL FA.

Purification: The sample was purified using a C18 solid-phase extraction (SPE) column. The SPE column was first activated with methanol and then equilibrated with 0.5% acetic acid. The sample was loaded onto the column, washed with 0.5% acetic acid, and finally eluted with 2 mL of ACN/0.5% acetic acid (60/40, *v*/*v*). The eluate was mixed thoroughly and then filtered through a 0.22 µm membrane for analysis.

### 2.4. Data Acquisition

#### 2.4.1. High-Resolution Mass (HRMS) Analysis

HRMS data of samples were acquired using Q Exactive HF-X (QE) (Thermo Fisher Scientific, Waltham, MA, USA) in Full Scan-ddMS2 mode for protein and peptide identification. The samples were separated on the chromatographic column (Hypersil GOLD C_18_ column (2.1 mm × 150 mm, 1.9 µm) (Thermo Fisher Scientific, Waltham, MA, USA)) using two mobile phases, A (0.1% FA in water) and B (0.1% FA in ACN), under a gradient elution program by Ultra Performance Liquid Chromatography (UPLC, Thermo Fisher Scientific, Waltham, MA, USA). The gradient was as follows: 0.0–0.2 min, 97–90% A; 0.2–16.0 min, 90–60% A; 16.0–17.0 min, 60–20% A; 17.0–17.5 min, 20% A; 17.5–18.5 min, 20–97% A; 18.5–25.0 min, 97% A. The flow rate was maintained at 0.2 mL/min, and the column temperature was set at 40 °C.

The mass conditions were as follows: spray voltage, 3400 V; capillary temperature, 320 °C; full scan resolution, 120,000 full-width at half maximum (FWHM); scanning quality range, 350–2000; automatic gain value, 3 × 106; secondary scanning, topN10; resolution, 30,000 FWHM; automatic gain value, 2 × 105; collision energy, 30 V.

#### 2.4.2. Liquid Chromatography-Tandem Mass (LC-MS/MS) Analysis

The mobile phase parameters for LC-MS/MS were consistent with those used in HRMS. LC-MS/MS conditions were as follows: spray voltage, 3500 V; sheath gas flow, 35 Arb; auxiliary gas flow, 15 Arb; capillary temperature, 275 °C; vaporizer temperature, 380 °C; acquisition cycle, 0.5 s; collision gas pressure, 1.5 mTorr; and Q1 and Q3 resolution, 0.7. Data analysis was conducted using TraceFinder (version 5.1, Thermo Fisher Scientific, Waltham, MA, USA).

### 2.5. Data Analysis

#### 2.5.1. Proteome Discoverer Software (PD) Analysis

The HRMS data were processed using PD software (version 2.2, Thermo Fisher Scientific, Waltham, MA, USA) to identify proteins and peptides. The PD software analysis involves two main steps: Processing and Consensus. For the Processing step, the following modifications were made in HT Process Parameters: the FASTA file from Uniprot website: http://www.uniprot.org (accessed on 9 January 2025) was uploaded to provide the reference protein database. The minimum peptide sequence length was set to 7, and the maximum length was set to 40. This adjustment ensures that peptides are neither too short (which could lead to incorrect protein matching) nor too long (which may hinder effective detection by mass).

The other default parameters of the software are sufficient to meet the requirements for proteomics analysis. The maximum number of missed cleavages was set to 2. Variable modifications included Oxidation (Methionine) and Acetylation (Protein N-term). Fixed modifications included Carbamidomethyl (Cysteine).

#### 2.5.2. Multivariate Statistical Analysis

Multivariate statistical analysis was performed using Wukong online tool powered by R language: https://www.omicsolution.com/wkomics/main/ (accessed on 12 March 2025), a comprehensive web-based tool designed for the analysis and visualization of omics data [29]. The Label-Free quantitative data of peptides was uploaded to the WuKong platform for clustering analysis. The visualized heatmaps and trend diagrams of categories were performed for facilitating subsequent peptide analysis.

## 3. Results and Discussion

### 3.1. Label-Free Quantitative Analysis

After extraction procedure, 10, 20, 50, 100, and 200 μL of the supernatant were pipetted, and the volume was made up to 200 μL with the extraction solution prior to adding DTT solution. Thus, the concentrations of the pork protein extracts were 5%, 10%, 25%, 50%, and 100% in sequence.

The protein and peptide data of the above varying-content pork samples was collected through HRMS, and Label-Free quantitative analysis was performed by PD. Finally, a total of 445 proteins and 3874 peptides were detected. For the authenticity analysis of meat products, the focus was on high-abundance proteins, which facilitates the widespread application of this method without the need for specialized pre-enrichment processes or advanced equipment. The peptides underwent primary filtering to exclude those with incomplete enzymatic digestion (residual enzymatic cleavage sites), and the remaining peptides were subjected to subsequent analysis.

### 3.2. Hierarchical Clustering Analysis

Multivariate statistical analysis is employed to process and interpret the complex proteomic data. Hierarchical clustering analysis (HCA) is performed to group peptides based on their response values across different sample concentrations. The resulting heatmap visualizes the clustering results, revealing which peptides exhibited similar trends in response values. This analysis helps to identify peptides with positive correlation to the concentration of the target meat, thereby narrowing down the list of potential target peptides for further validation.

**Figure 1:**
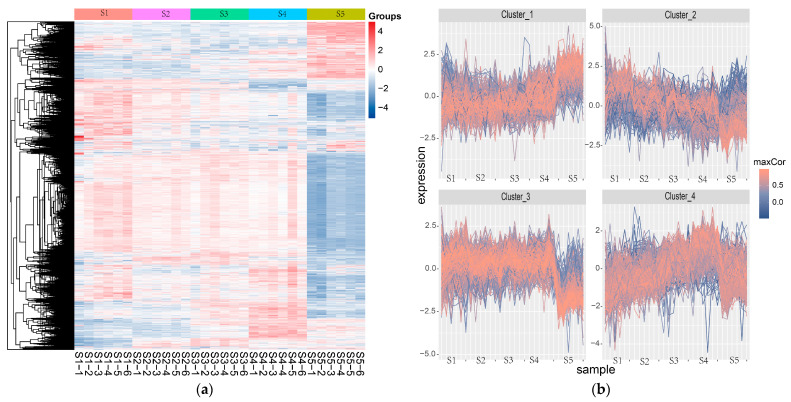
Hierarchical clustering analysis of different content pig meat: heatmap (**a**) and trend diagram (**b**).

The quantitative data of the peptides obtained by PD were subjected to preprocessing steps. Missing values were imputed using the K-nearest neighbors (KNN) method with a proportion of 0.5 and a neighborhood size of 5 in an overall-consideration manner. This method helped to fill in the missing data based on the values of similar data, thereby preserving the overall structure of the dataset. The data was then standardized through both row and column normalization to ensure that the data was on a comparable scale, which is essential for accurate clustering and pattern recognition. Hierarchical clustering analysis was performed, and the results are shown in Figure 1.

In Figure 1, S1–S5 represent the data collected from protein extract samples with the concentrations of 5%, 10%, 25%, 50%, and 100%, respectively. In this study, each concentration of the sample was tested in six biological replicates. The peptides were grouped into four groups. The heatmap (Figure 1a) provides a visual representation of the peptide trends at different concentrations. From the visualization trend diagram (Figure 1b), the trends exhibited by the four clusters differ with varying concentration. The peptides in clusters 1 and 4 showed an increasing trend with the increase in sample concentration, which is consistent with the principle of quantitative analysis. Therefore, 795 peptides from these two clusters were selected, excluding 80% of the peptides and significantly reducing the workload.

To confirm the species-specificity of the peptides, all 795 screened peptides were subjected to BLAST analysis on the UniProt website: https://www.uniprot.org/blast (accessed on 6 May 2025), searching the entire database, with retention of peptides exhibiting 100% identity and being exclusively derived from pig. Some peptides from uncommon species, such as bacteria, were also retained because they do not interfere with the authenticity assessment of routine meat products. Following BLAST analysis, 89 pig-specific peptides were selected, ensuring no cross-reactivity with common meat species.

### 3.3. Screening of Target Quantitative Peptides by PRM Method

PRM is a targeted, high-resolution and high-sensitivity method that allows for the selective monitoring and quantitative determination of specific peptides. The PRM method is straightforward to establish. Simply input the precursor ion mass-to-charge ratio (*m*/*z*) and charge of the peptides (which can be identified through analysis in the aforementioned PD analysis, with precursor ions exhibiting the highest response values), selected to be measured into the inclusion list, and the measurement can be carried out. The mass spectrometry parameters used for PRM were the same as those for Full Scan-ddMS2. The solutions with different concentrations were detected by the PRM in HRMS.

The PRM data of the 89 target peptides were imported into Skyline software (version 21.1, MacCoss Lab, University of Washington, VA, USA), a powerful tool for building and analyzing targeted proteomics assays. In Skyline software, the retention time of the same peptide in different samples was compared to different data to ensure the uniformity of peptide response values integration. Then, the linear relationship of the peptides at different concentrations was examined. Finally, 17 peptides exhibiting strong linear relationships (correlation coefficients greater than 0.99) were identified. The information of these 17 peptides is shown in Table 1. The high-resolution mass spectrometry of 17 peptides is provided in the Supplementary File S1. Among them, three peptides previously identified for accurate quantification (Pep2 GGPLTAAYR, Pep3 HDPSLLPWTASYDPGSAK, and Pep7 EPITVSSDQMAK) [28] were also found in this study, confirming the effectiveness of the screening method in improving the efficiency and accuracy of quantitative peptide selection.

### 3.4. Validation of Target Quantitative Peptides by LC-MS/MS

The multiple reaction monitoring (MRM) mode of LC-MS/MS is capable of qualitative and quantitative analysis by measuring specific product ions derived from specific precursor ions, effectively reducing matrix interference and improving analytical selectivity. Compared to HRMS, LC-MS/MS has stronger anti-matrix interference ability, higher quantitative accuracy, and is also more cost-effective and thus more suitable for the widespread application of authenticity assessment in food analysis.

#### 3.4.1. Development of LC-MS/MS Method

**Figure 2:**
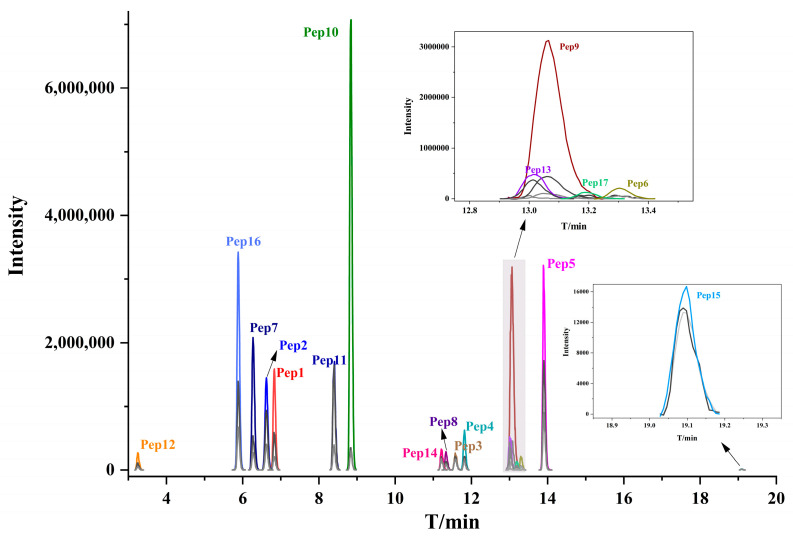
The chromatogram of each peptide marker detected by LC-MS/MS.

To construct the LC-MS/MS method, the PRM mass spectrometry of the 17 quantifiable and species-specific peptides were reviewed using Xcalibur software (version 1.6, Thermo Fisher Scientific, Waltham, MA, USA). For each peptide, the top seven fragment ions are selected based on intensity to generate MRM ion information, which was then imported into the LC-MS/MS system. Due to differences in working principles, resolution, and sensitivity between LC-MS/MS and HRMS, the response of peptide fragment ions may vary in LC-MS/MS. Therefore, multiple fragment ions were chosen to ensure reliable detection and quantification. The final MRM method was established by retaining the three fragment ions with the highest intensity and minimal matrix interference, selected from the spectra acquired through LC-MS/MS analysis. The chromatogram of 17 peptides extracted from LC-MS/MS is represented in Figure 2.

#### 3.4.2. Validation by Quantitative Standard Curve

To evaluate the matrix interference and accuracy of the target quantitative peptides, standard curves were constructed using protein extracts from both pork and beef. Different volumes of pork protein extract supernatant (10 μL, 20 μL, 50 μL, 100 μL, and 190 μL) were mixed with beef protein extract supernatant to make up to 200 μL, followed by enzymatic hydrolysis and purification. The mass concentration of pork was calculated as 0.5 mg/mL, 1 mg/mL, 2.5 mg/mL, 5 mg/mL, and 9.5 mg/mL. The ion response intensity of the target quantitative peptides was plotted against the mass concentration of pork to obtain the standard curves.

The selection of suitable target quantitative peptides should consider factors such as high specificity, good linearity, high sensitivity, good stability, and high accuracy [17,30,31]. The linear fitting analysis of the 17 peptides showed that six peptides met the screening criteria for target quantitative peptides (R^2^ greater than 0.99). The linear equations of these six peptides are listed in Table 2. Among them, GGPLTAAYR and HDPSLLPWTASYDPGSAK had been previously validated for their accuracy in pork quantification in our earlier research [28]. The peptides TLAFLFAER and TVLGNFAAFVQK have been utilized for pork determination in several studies. Christoph von Bargen [32] selected TLAFLFAER as a marker peptide to detect pork in a beef matrix system. Magdalena Montowska [33] showed a novel and rapid LESA-MS method to identify beef, pork, horse, chicken, and turkey species in processed meat products by 25 heat stable peptide markers, among which the peptide TLAFLFAER was a pig-specific peptide. S.A. Sarah [34] used LC-QTOF-MS to detect porcine-specific peptide markers (TVLGNFAAFVQK) from thermally processed meat that could differentiate pork from beef, chevon, and chicken meat.

#### 3.4.3. Method Verification

**Figure 3:**
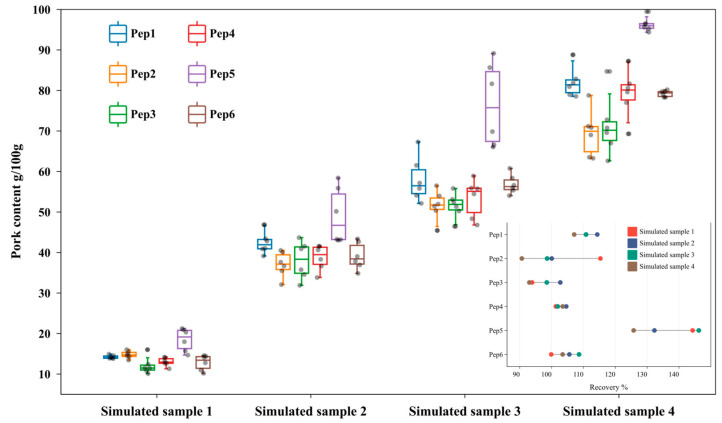
Pork contents and recovery results determined by the six peptides in simulated samples (n = 6). Each box delineates the first quartile (Q1), median, and third quartile (Q3).

The six target quantitative peptides were used to determine the pork content in the simulated meatball samples (12.75 g/100 g, 36.95 g/100 g, 52.24 g/100 g, and 76.45 g/100 g). The results were compared with the known true content of pork to evaluate the accuracy of the method. The pork contents and recovery rates of the six peptides in the simulated samples are shown in Figure 3. The recovery rates of the peptides, except Pep5, were mostly concentrated between 78% and 128%, with deviations less than 12%. Pep5 had recovery rates ranging from 114% to 150%, with deviations less than 13%. The elevated recovery of Pep5 may be attributed to ion enhancement effects in complex matrices, so it could be used as a backup peptide. Therefore, using the peptides screened in this study, the pork content in the samples can be accurately calculated.

Additionally, species specificity was an important factor affecting the accuracy of quantitative results. For pure pork samples, the previously identified peptides Pep2, Pep3, and Pep7 [28] were used for accurate quantification. However, in the study, Pep7 EPITVSSDQMAK showed lower quantification results due to its lack of pork specificity. It was also identified in cattle, duck, and chicken in the Blast analysis and could be only used for quantitative analysis in pure pork samples. The peptides Pep2 to Pep6 identified in this study were all specific peptides, while Pep1 was a relatively specific peptide because it was only found in pig and bacteria origin in the Blast analysis and could be used as an auxiliary target quantitative peptide.

## 4. Conclusions

This study presents a rapid and efficient method for screening target quantitative peptides. By combining high-resolution mass spectrometry with multivariate statistical analysis, 80% of ineffective peptides were excluded. Further Blast analysis confirmed the specificity of the peptides. Through the construction of quantitative standard curves and linear fitting, five specific target quantitative peptides were identified that can accurately quantify the pork content in samples. Compared to the existing methods that randomly screen quantitative peptides from qualitative analysis data, this method significantly enhances the efficiency and accuracy of peptide screening for meat authentication.

Nevertheless, targeted peptide quantification by MS confronts persisting methodological challenges, including insufficient standardization due to undefined reference standards; content variations of proteins/peptides across tissue types, geographical origins, and species strains; ambiguous mass conversion factors between peptides and meat matrices; and the uncharacterized impacts of processing techniques on protein and peptide. Addressing these knowledge gaps necessitates targeted experimental investigations, where multi-technique approaches may improve quantification accuracy.

## Figures and Tables

**Table 1:** Information of 17 peptides.

Peptide ID	Peptide Sequence	Peptide Length	*m*/*z*	Charge	RT/ min	UniProt Accession Number	Protein Source
Pep1	VIFADGSR	8	432.733	2	5.90	A0A480K2R3	phosphoglucomutase (alpha-D-glucose-1,6-bisphosphate-dependent)
Pep2	GGPLTAAYR	9	453.246	2	6.63	Q5S1S4	Carbonic anhydrase 3
Pep3	HDPSLLPWTASYDPGSAK	18	647.983	3	11.59	Q5S1S4	Carbonic anhydrase 3
Pep4	ADAIGLSLIK	10	500.805	2	11.80	A0A4X1WCQ0	Glycerol-3-phosphate dehydrogenase
Pep5	TLAFLFAER	9	534.298	2	13.89	A0A8D1T8Y2	Myosin-4
Pep6	TVLGNFAAFVQK	12	647.861	2	13.32	A0A287BAY9	Albumin
Pep7	EPITVSSDQMAK	12	653.322	2	6.28	Q5S1S4	Carbonic anhydrase 3
Pep8	HFLEELLTTQCDR	13	831.405	2	11.35	A0A8D1S3B2	Myosin light chain
Pep9	HPDGVAVVGIFLK	13	676.391	2	13.14	Q5S1S4	Carbonic anhydrase 3
Pep10	IVTDLAK	7	380.234	2	8.84	A0A287BAY9	Albumin
Pep11	NQMEIGEDPK	10	580.766	2	8.42	A0A4X1VUZ8	Myosin binding protein C, fast type
Pep12	SALAHAVQSSR	11	376.203	3	3.12	A0A8D1T8Y2	Myosin-4
Pep13	TIVPGNIFK	9	494.795	2	13.04	A0A480SN35	Titin
Pep14	ADISSFVIESAER	13	712.357	2	11.24	A0A4X1VUZ8	Myosin binding protein C, fast type
Pep15	GPGTSFEFALAIVEALAGK	19	939.505	2	19.10	A0A8D0IT98	protein deglycase
Pep16	ILVDEER	7	437.238	2	6.83	A0A8D1BMD0	Alpha-1,4 glucan phosphorylase
Pep17	MAEILSGTETVSLTHVAQEALR	22	786.078	3	13.18	A0A480SN35	Titin

**Table 2:** Information and linear equations of six target quantitative peptides.

Peptide ID	*m*/*z*	Product Ions (*m*/*z*)	Linear Equation	R^2^
Pep1	432.733	652.304/213.160/505.236	y = 201,843x − 28,140	0.9913
Pep2	453.246	581.304/480.256/212.103	y = 209,641x − 28,045	0.9926
Pep3	647.983	550.262/824.379/895.416	y = 27,132x + 1769.7	0.9937
Pep4	500.805	630.418/258.108/743.500	y = 194,145x − 23,236	0.9938
Pep5	534.298	853.456/215.139/286.176	y = 512,434x − 74,435	0.9935
Pep6	647.861	201.123/592.347/374.239	y = 69,530x − 7210.3	0.9979

## Data Availability

The original contributions presented in the study are included in the article. Further inquiries can be directed to the corresponding author.

## Supplementary Materials

The following supporting information can be downloaded at https://www.mdpi.com/article/10.3390/foods14173048/s1: File S1: High-resolution mass spectrometry of 17 peptides.

## References

[1] Farag M.R., Alagawany M., Elgammal M., Tiwari R., Dhama K. (2015). Identification of Different Animal Species in Meat and Meat Products-Trends and Advances. Adv. Anim. Vet. Sci..

[2] Su G., Yu C., Liang S., Wang W., Wang H. (2024). Multi-omics in food safety and authenticity in terms of food components. Food Chem..

[3] Qamar Z., Mohammad A., Khairil M.N.F., Raja N.R.M.H., Irwan H. (2020). Current Analytical Methods for Porcine Identification in Meat and Meat Products. Food Chem..

[4] Kamruzzaman M., Makino Y., Oshita S., Liu S. (2015). Assessment of Visible Near-Infrared Hyperspectral Imaging as a Tool for Detection of Horsemeat Adulteration in Minced Beef. Food Bioprocess Technol..

[5] Chen Z., Wu T., Xiang C., Xu X., Tian X. (2019). Rapid Identification of Rainbow Trout Adulteration in Atlantic Salmon by Raman Spectroscopy Combined with Machine Learning. Molecules.

[6] Bergana M.M., Adams K.M., Harnly J., Moore J.C., Xie Z. (2019). Non-targeted Detection of Milk Powder Adulteration by 1H NMR spectroscopy and Conformity Index Analysis. J. Food Compos. Anal..

[7] Sikorska E., Khmelinskii I., Sikorski M. (2019). Fluorescence spectroscopy and imaging instruments for food quality evaluation. Eval. Technol. Food Qual..

[8] At-Kaddour A., Loudiyi M., Ferlay A., Gruffat D. (2018). Performance of fluorescence spectroscopy for beef meat authentication: Effect of excitation mode and discriminant algorithms. Meat Sci..

[9] Velioglu H.M., Sezer B., Bilge G., Baytur S.E., Boyaci I.H. (2018). Identification of offal adulteration in beef by laser induced breakdown spectroscopy (LIBS). Meat Sci..

[10] Al-Sarayreh M., Reis M.M., Yan W.Q., Klette R. (2020). Potential of deep learning and snapshot hyperspectral imaging for classification of species in meat. Food Control.

[11] Hassoun A., Måge I., Schmidt W.F., Temiz H.T., Li L., Kim H.-Y., Nilsen H., Biancolillo A., Aït-Kaddour A., Sikorski M. (2020). Fraud in Animal Origin Food Products: Advances in Emerging Spectroscopic Detection Methods over the Past Five Years. Foods.

[12] Al-Kahtani H.A., Ismail E.A., Asif Ahmed M. (2017). Pork detection in binary meat mixtures and some commercial food products using conventional and real-time PCR techniques. Food Chem..

[13] Cottenet G., Blancpain C., Chuah P.F., Cavin C. (2020). Evaluation and application of a next generation sequencing approach for meat species identification. Food Control.

[14] Takasaki K., Yamakoshi Y., Futo S. (2018). Single-Laboratory Validation of Rapid and Easy DNA Strip for Porcine DNA Detection in Beef Meatballs. J. AOAC Int..

[15] Muflihah, Hardianto A., Kusumaningtyas P., Prabowo S., Hartati Y.W. (2023). DNA-based detection of pork content in food. Heliyon.

[16] Perestam A.T., Fujisaki K.K., Nava O., Hellberg R.S. (2017). Comparison of real-time PCR and ELISA-based methods for the detection of beef and pork in processed meat products. Food Control.

[17] Kim G.-D., Seo J.-K., Yum H.-W., Jeong J.-Y., Yang H.-S. (2017). Protein markers for discrimination of meat species in raw beef, pork and poultry and their mixtures. Food Chem..

[18] Hafner L., Kalkhof S., Jira W. (2021). Authentication of nine poultry species using high-performance liquid chromatography-tandem mass spectrometry. Food Control.

[19] Stachniuk A., Sumara A., Montowska M., Fornal E. (2019). Liquid chromatography–mass spectrometry bottom-up proteomic methods in animal species analysis of processed meat for food authentication and the detection of adulterations. Mass Spectrom. Rev..

[20] Zhang M., Li Y., Zhang Y., Kang C., Zhao W., Ren N., Guo W., Wang S. (2022). Rapid LC-MS/MS method for the detection of seven animal species in meat products. Food Chem..

[21] Magdalena M., Emilia F. (2019). Absolute quantification of targeted meat and allergenic protein additive peptide markers in meat products. Food Chem..

[22] Stachniuk A., Trzpil A., Czeczko R., Nowicki L., Ziomkowska M., Fornal E. (2024). Absolute quantification of targeted rabbit liver- and meat tissue-specific peptide markers in highly processed food products. Food Chem..

[23] Barbara P., Martina V., Andrea F., Francesca L., Michele S., Andrea L., Tullia T., Stefano S. (2019). Species specific marker peptides for meat authenticity assessment: A multispecies quantitative approach applied to Bolognese sauce. Food Control.

[24] Sentandreu M.A., Fraser P.D., Halket J., Patel R., Bramley P.M. (2010). A Proteomic-Based Approach for Detection of Chicken in Meat Mixes. J. Proteome Res..

[25] Windarsih A., Suratno, Warmiko H.D., Indrianingsih A.W., Rohman A., Ulumuddin Y.I. (2022). Untargeted metabolomics and proteomics approach using liquid chromatography-Orbitrap high resolution mass spectrometry to detect pork adulteration in Pangasius hypopthalmus meat. Food Chem..

[26] Pu K., Qiu J., Tong Y., Liu B., Cheng Z., Chen S., Ni W., Lin Y., Ng K.M. (2023). Integration of Non-targeted Proteomics Mass Spectrometry with Machine Learning for Screening Cooked Beef Adulterated Samples. J. Agric. Food Chem..

[27] Zhang Y., Liu M., Wang S., Kang C., Zhang M., Li Y. (2022). Identification and quantification of fox meat in meat products by liquid chromatography-tandem mass spectrometry. Food Chem..

[28] Li Y., Zhang Y., Kang C., Zhao W., Li S., Wang S. (2021). Assessment of carbonic anhydrase 3 as a marker for meat authenticity and performance of LC-MS/MS for pork content. Food Chem..

[29] Wang G., Zhou G., Ren H.-w., Xu Y., Yang Y., Guo L., Liu N. (2018). Peptide biomarkers identified by LC–MS in processed meats of five animal species. J. Food Compos. Anal..

[30] Nalazek-Rudnicka K., Kłosowska-Chomiczewska I., Wasik A., Macierzanka A. (2019). MRM-MS of marker peptides and their abundance as a tool for authentication of meat species and meat cuts in single-cut meat products. Food Chem..

[31] Claydon A.J., Grundy H.H., Charlton A.J., Romero M.R. (2015). Identification of novel peptides for horse meat speciation in highly processed foodstuffs. Food Addit. Contam..

[32] Bargen C.v., Brockmeyer J., Humpf H.-U. (2014). Meat authentication: A new HPLC-MS/MS based method for the fast and sensitive detection of horse and pork in highly processed food. J. Agric. Food Chem..

[33] Montowska M., Alexander M.R., Tucker G.A., Barrett D.A. (2015). Authentication of processed meat products by peptidomic analysis using rapid ambient mass spectrometry. Food Chem..

[34] Sarah S.A., Faradalila W.N., Salwani M.S., Amin I., Karsani S.A., Sazili A.Q. (2016). LC-QTOF-MS identification of porcine-specific peptide in heat treated pork identifies candidate markers for meat species determination. Food Chem..

