# Supplementary figures and images for: Visualization-Based Rapid Screening and Quantitative Analysis of Target Peptides for Meat Authentication
Source: Foods. 2025 Aug 29;14(17):3048. doi: 10.3390/foods14173048 (PMC12428751; doi:10.3390/foods14173048)

# Supplementary materials: High-resolution mass spectrometry of 17 peptides

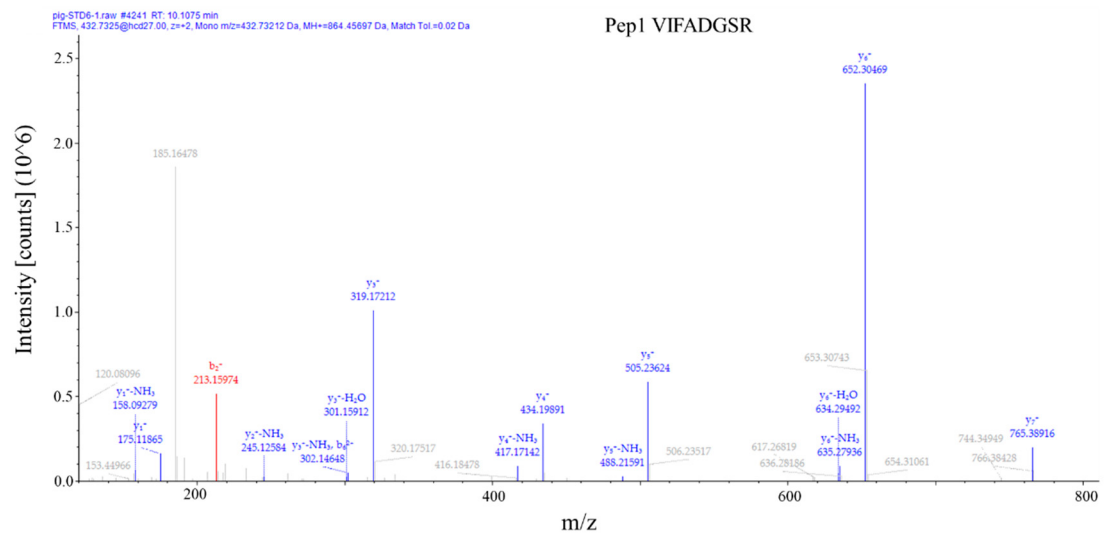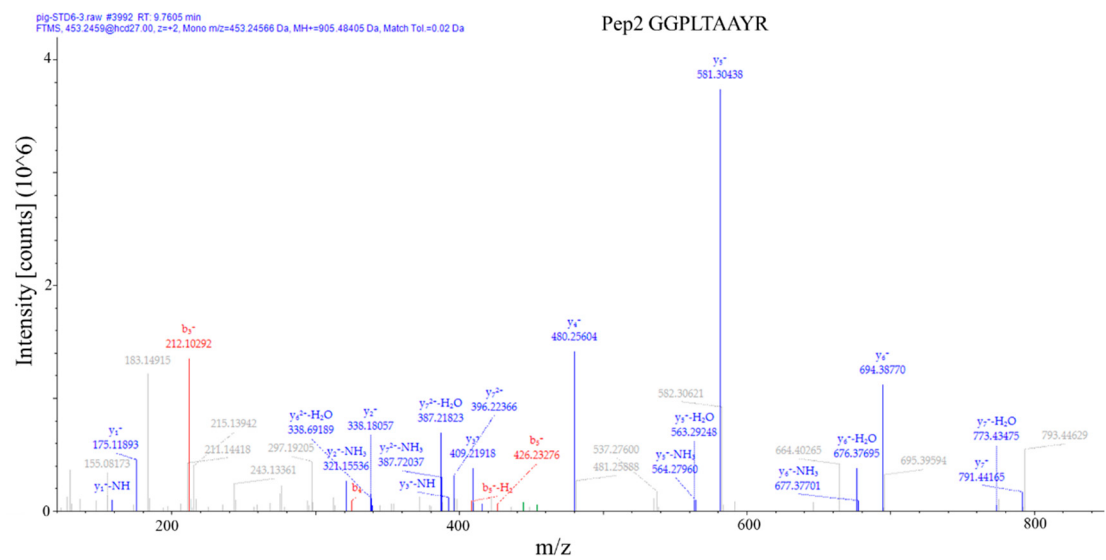

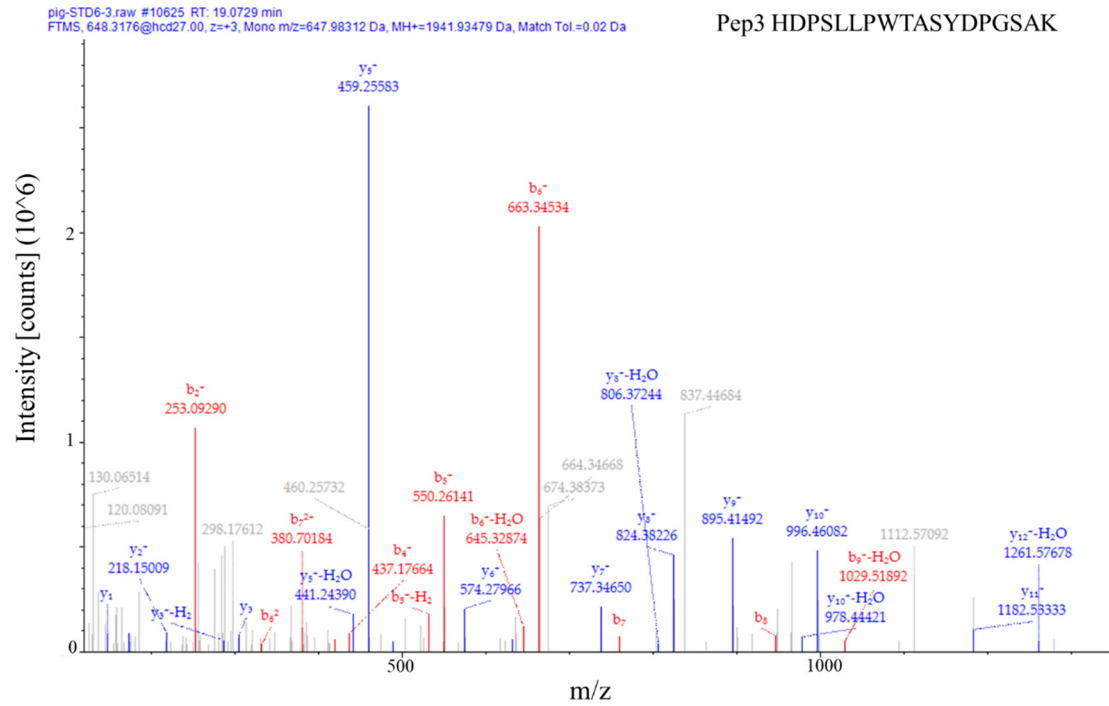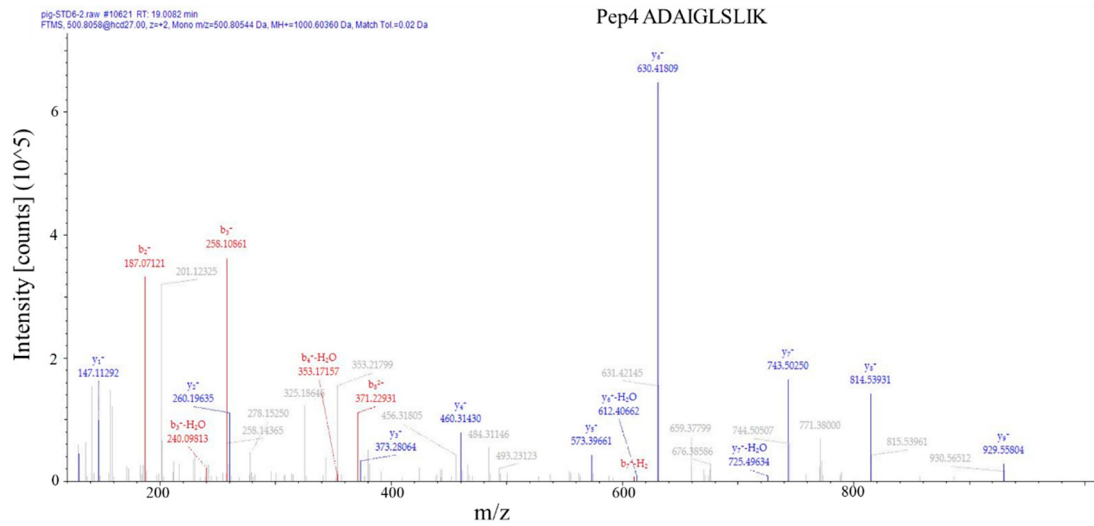

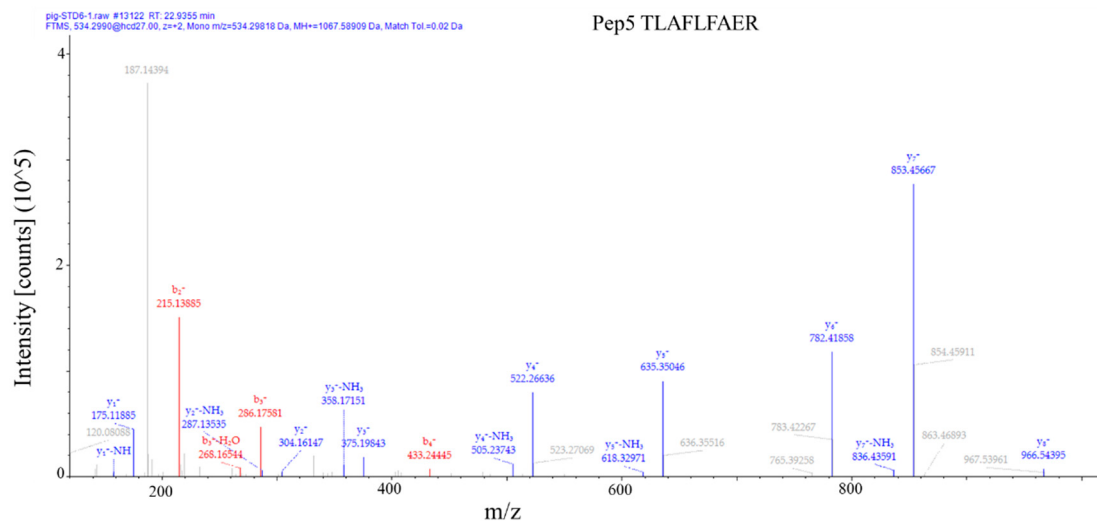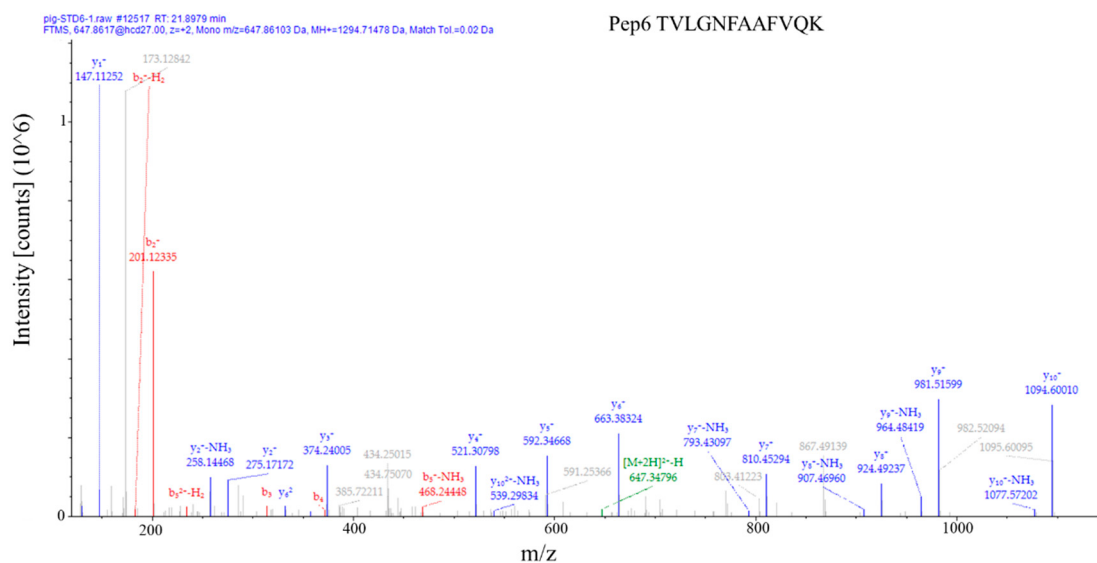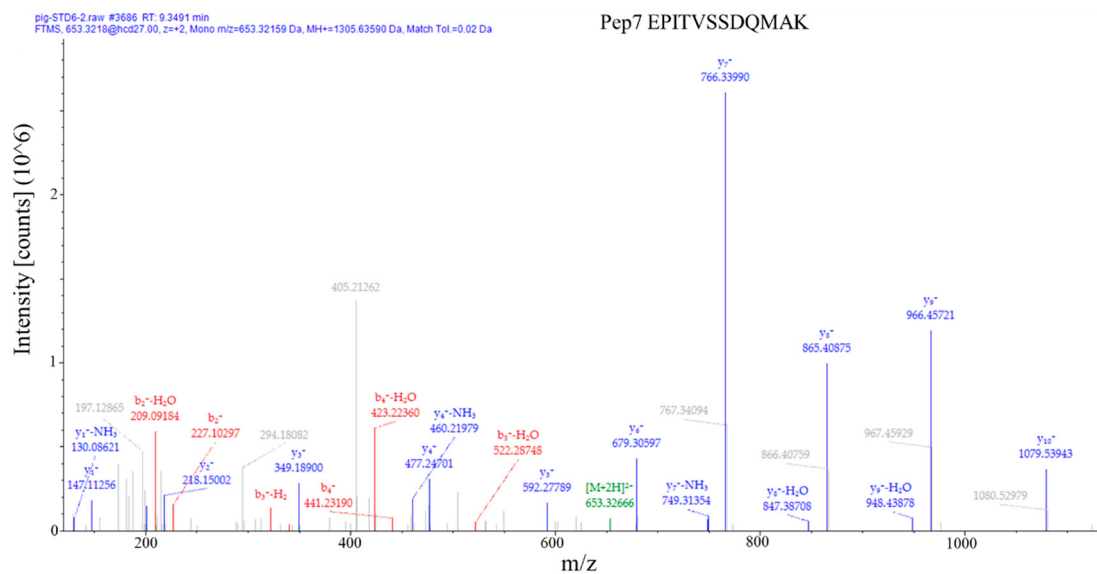

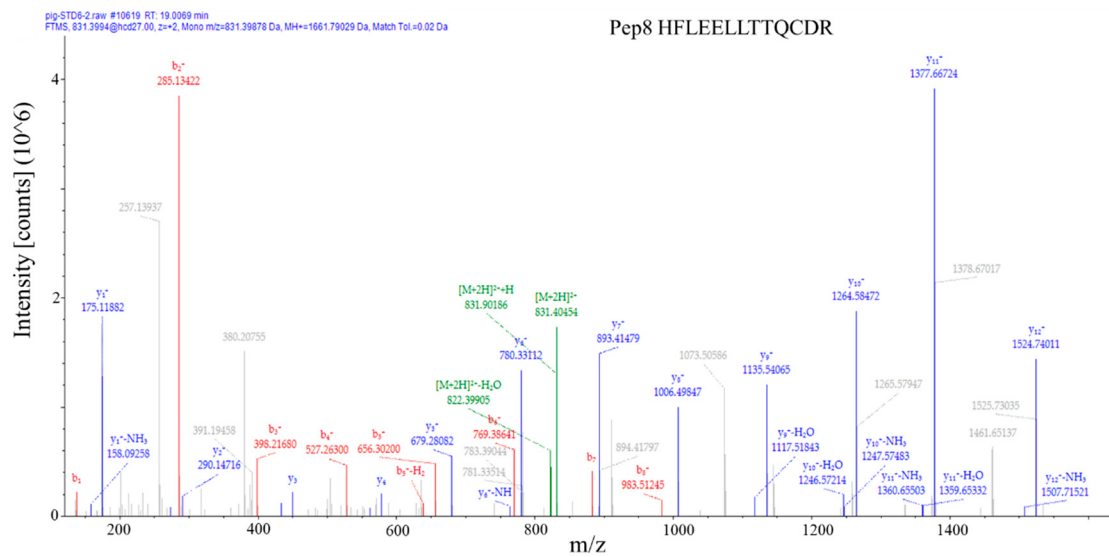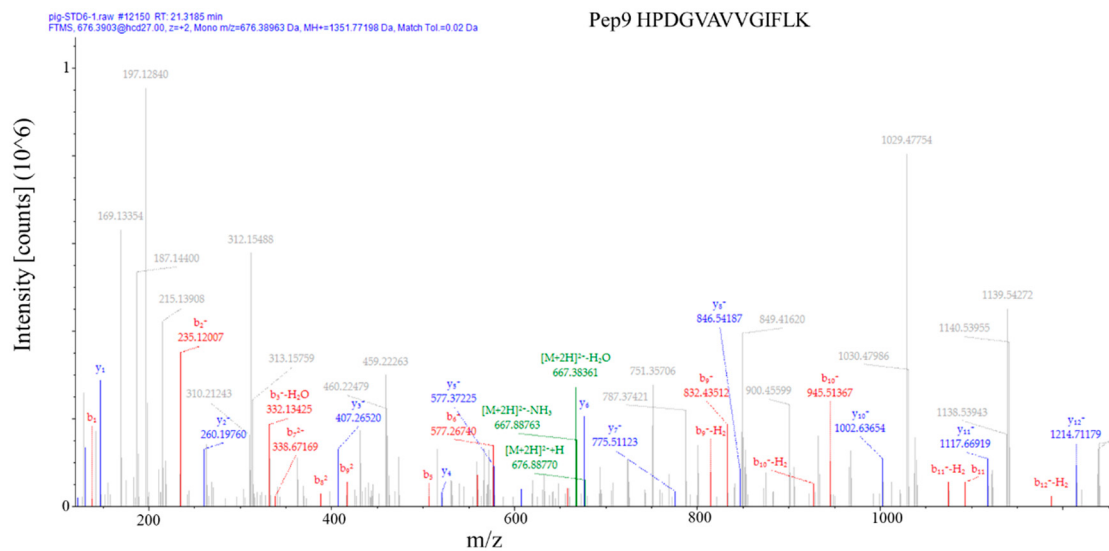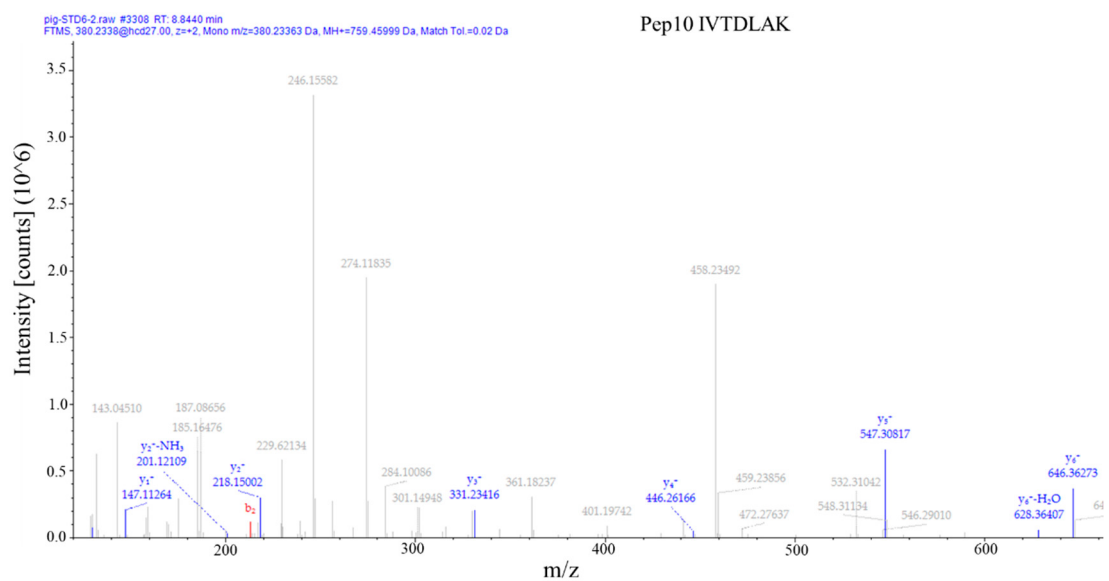

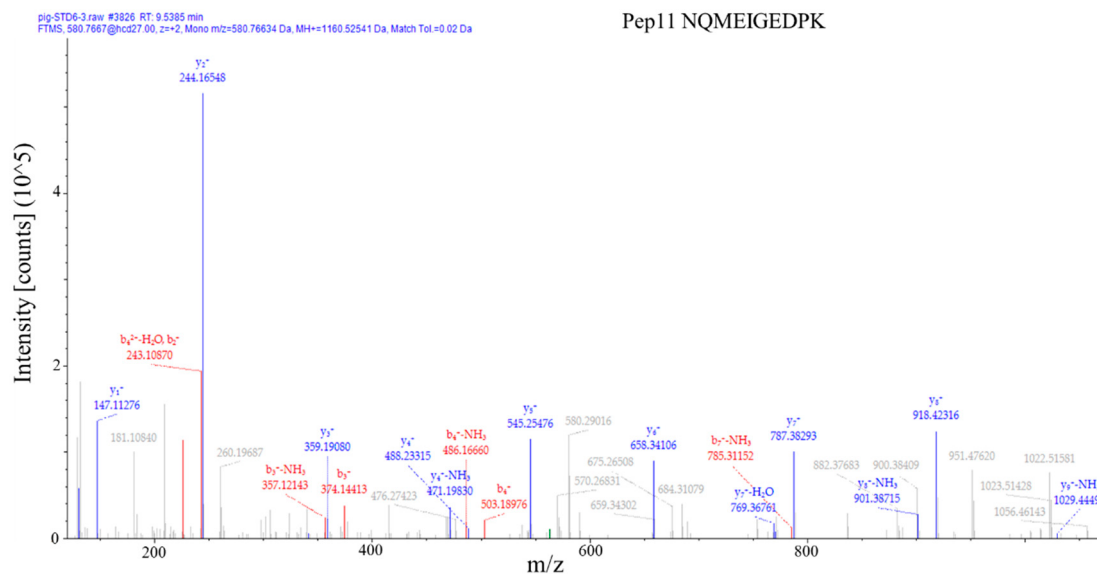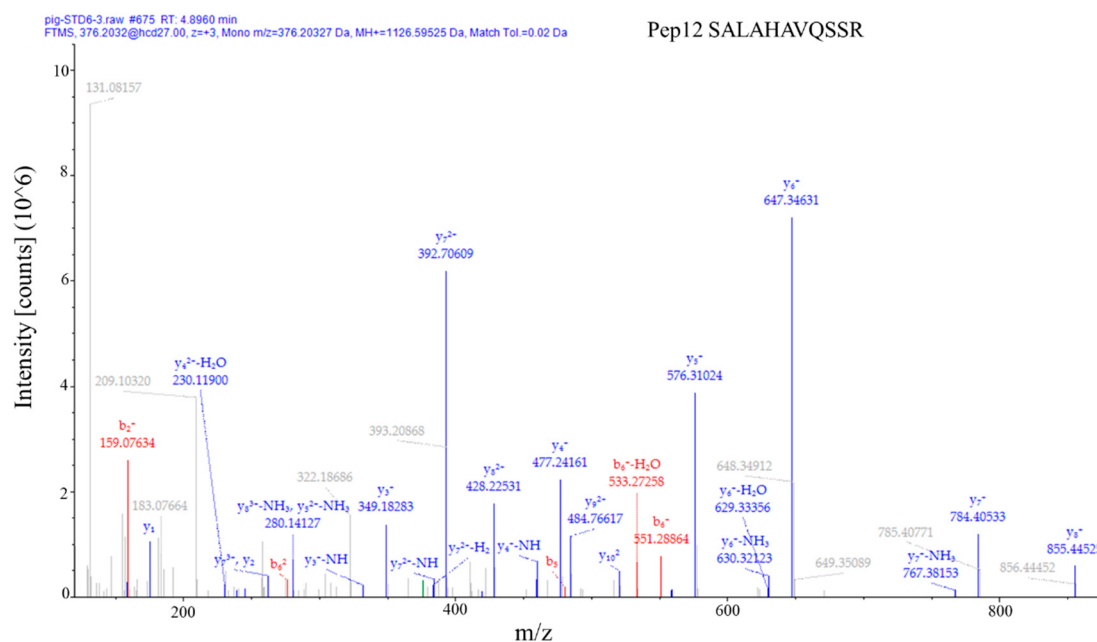

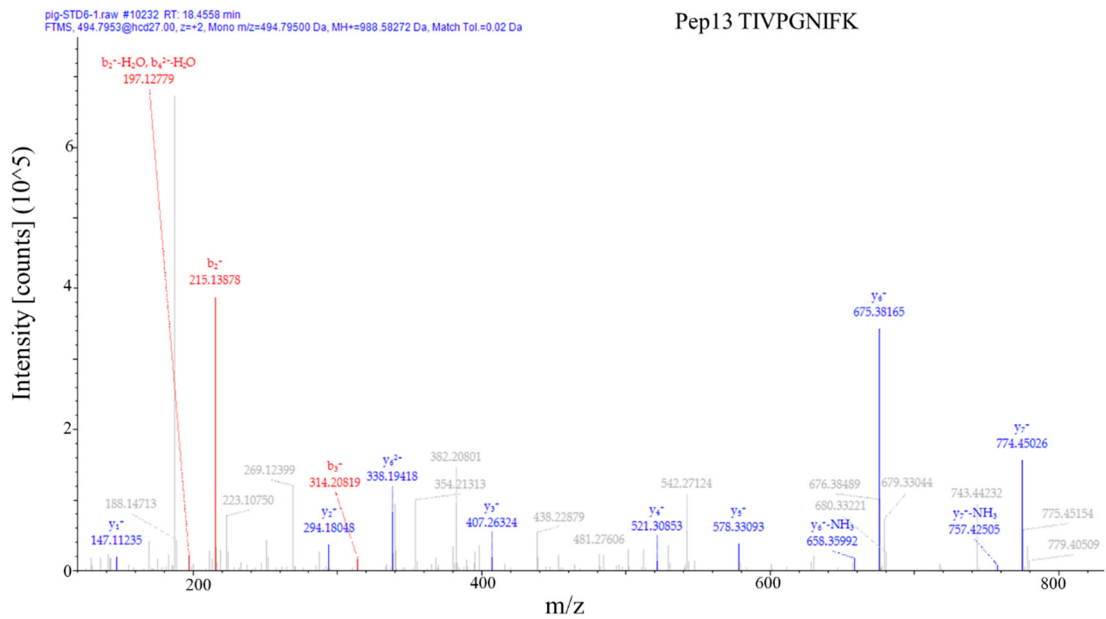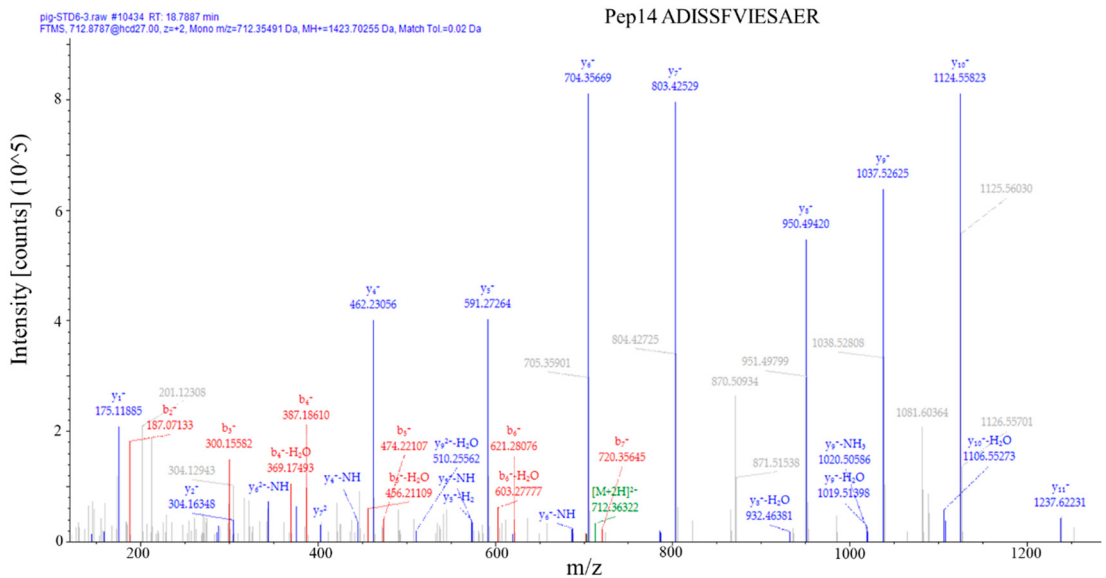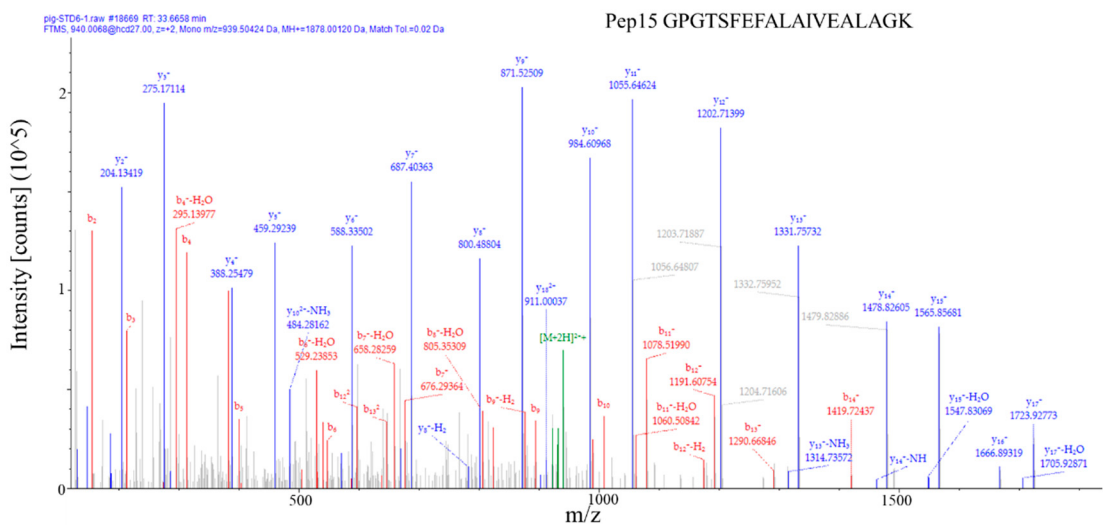

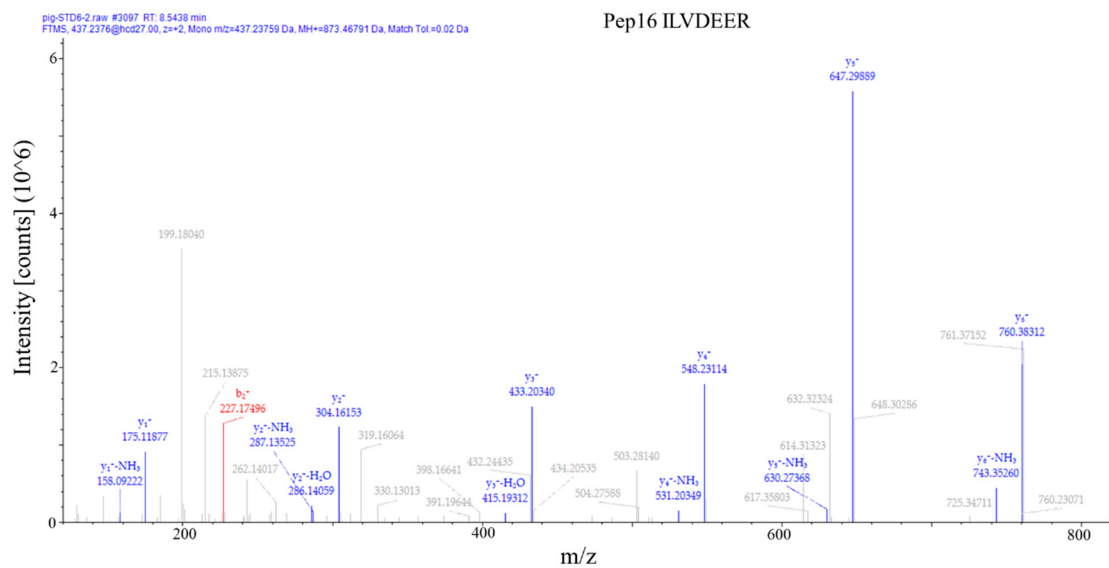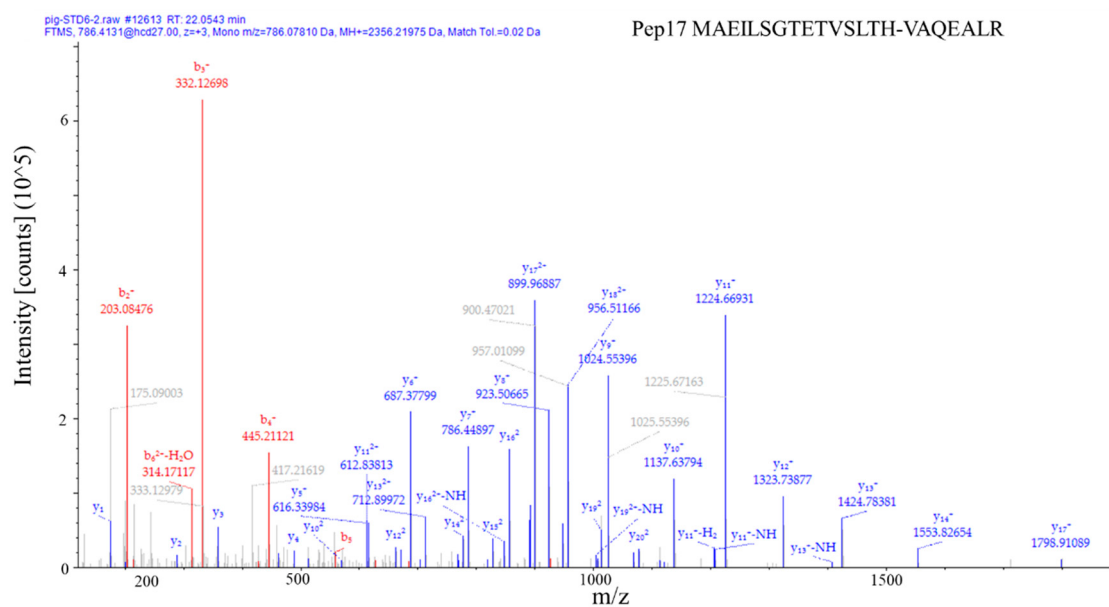

Supplement: Supplementary file 1 [file foods-14-03048-s001.zip › foods-3770511-supplementary.pdf]
